# Role of self-care in management of diabetes mellitus

**DOI:** 10.1186/2251-6581-12-14

**Published:** 2013-03-05

**Authors:** Saurabh RamBihariLal Shrivastava, Prateek Saurabh Shrivastava, Jegadeesh Ramasamy

**Affiliations:** Department of Community Medicine, Shri Sathya Sai Medical College & Research Institute, Ammapettai village, Thiruporur - Guduvancherry Main Road, Sembakkam Post, 603108 Kancheepuram, Tamil Nadu India

**Keywords:** Diabetes, Self-care, Compliance, Physical activity, Lifestyle modification

## Abstract

Diabetes mellitus (DM) is a chronic progressive metabolic disorder characterized by hyperglycemia mainly due to absolute (Type 1 DM) or relative (Type 2 DM) deficiency of insulin hormone. World Health Organization estimates that more than 346 million people worldwide have DM. This number is likely to more than double by 2030 without any intervention. The needs of diabetic patients are not only limited to adequate glycemic control but also correspond with preventing complications; disability limitation and rehabilitation. There are seven essential self-care behaviors in people with diabetes which predict good outcomes namely healthy eating, being physically active, monitoring of blood sugar, compliant with medications, good problem-solving skills, healthy coping skills and risk-reduction behaviors. All these seven behaviors have been found to be positively correlated with good glycemic control, reduction of complications and improvement in quality of life. Individuals with diabetes have been shown to make a dramatic impact on the progression and development of their disease by participating in their own care. Despite this fact, compliance or adherence to these activities has been found to be low, especially when looking at long-term changes. Though multiple demographic, socio-economic and social support factors can be considered as positive contributors in facilitating self-care activities in diabetic patients, role of clinicians in promoting self-care is vital and has to be emphasized. Realizing the multi-faceted nature of the problem, a systematic, multi-pronged and an integrated approach is required for promoting self-care practices among diabetic patients to avert any long-term complications.

## Introduction

Diabetes mellitus (DM) is a chronic progressive metabolic disorder characterized by hyperglycemia mainly due to absolute (Type 1 DM) or relative (Type 2 DM) deficiency of insulin hormone[1]. DM virtually affects every system of the body mainly due to metabolic disturbances caused by hyperglycemia, especially if diabetes control over a period of time proves to be suboptimal[1]. Until recently it was believed to be a disease occurring mainly in developed countries, but recent findings reveal a rise in number of new cases of type 2 DM with an earlier onset and associated complications in developing countries[2–4]. Diabetes is associated with complications such as cardiovascular diseases, nephropathy, retinopathy and neuropathy, which can lead to chronic morbidities and mortality[5, 6]. World Health Organization (WHO) estimates that more than 346 million people worldwide have DM. This number is likely to more than double by 2030 without any intervention. Almost 80% of diabetes deaths occur in low and middle-income countries[7]. According to WHO report, India today heads the world with over 32 million diabetic patients and this number is projected to increase to 79.4 million by the year 2030[8]. Recent surveys indicate that diabetes now affects a staggering 10-16% of urban population and 5-8% of rural population in India and Sri Lanka[9–11].

### Addressing needs of diabetic patients

One of the biggest challenges for health care providers today is addressing the continued needs and demands of individuals with chronic illnesses like diabetes[12]. The importance of regular follow-up of diabetic patients with the health care provider is of great significance in averting any long term complications. Studies have reported that strict metabolic control can delay or prevent the progression of complications associated with diabetes[13, 14]. The needs of diabetic patients are not only limited to adequate glycemic control but also correspond with preventing complications; disability limitation and rehabilitation. Some of the Indian studies revealed very poor adherence to treatment regimens due to poor attitude towards the disease and poor health literacy among the general public[15, 16]. The introduction of home blood glucose monitors and widespread use of glycosylated hemoglobin as an indicator of metabolic control has contributed to self-care in diabetes and thus has shifted more responsibility to the patient[17, 18]. In a study done in Scotland, it was suggested that the role of the health professional is crucial to patient’s understanding of their blood glucose fluctuations with an appropriate self-care action[19].

### Self-care in diabetes

Self-care in diabetes has been defined as an evolutionary process of development of knowledge or awareness by learning to survive with the complex nature of the diabetes in a social context[20, 21]. Because the vast majority of day-to-day care in diabetes is handled by patients and/or families[22], there is an important need for reliable and valid measures for self-management of diabetes[23–25]. There are seven essential self-care behaviors in people with diabetes which predict good outcomes. These are healthy eating, being physically active, monitoring of blood sugar, compliant with medications, good problem-solving skills, healthy coping skills and risk-reduction behaviors[26]. These proposed measures can be useful for both clinicians and educators treating individual patients and for researchers evaluating new approaches to care. Self-report is by far the most practical and cost-effective approach to self-care assessment and yet is often seen as undependable. Diabetes self-care activities are behaviors undertaken by people with or at risk of diabetes in order to successfully manage the disease on their own[26]. All these seven behaviors have been found to be positively correlated with good glycemic control, reduction of complications and improvement in quality of life[27–31]. In addition, it was observed that self-care encompasses not only performing these activities but also the interrelationships between them[32]. Diabetes self-care requires the patient to make many dietary and lifestyle modifications supplemented with the supportive role of healthcare staff for maintaining a higher level of self-confidence leading to a successful behavior change[33].

### Diabetes self management education

Though genetics play an important role in the development of diabetes, monozygotic twin studies have certainly shown the importance of environmental influences[34]. Individuals with diabetes have been shown to make a dramatic impact on the progression and development of their disease by participating in their own care[13]. This participation can succeed only if those with diabetes and their health care providers are informed about taking effective care for the disease. It is expected that those with the greatest knowledge will have a better understanding of the disease and have a better impact on the progression of the disease and complications.

The American Association of Clinical Endocrinologists emphasizes the importance of patients becoming active and knowledgeable participants in their care[35]. Likewise, WHO has also recognized the importance of patients learning to manage their diabetes[36]. The American Diabetes Association had reviewed the standards of diabetes self management education and found that there was a four-fold increase in diabetic complications for those individuals with diabetes who had not received formal education concerning self-care practices[37]. A meta-analysis of self-management education for adults with type-2 diabetes revealed improvement in glycemic control at immediate follow-up. However, the observed benefit declined one to three months after the intervention ceased, suggesting that continuing education is necessary[38]. A review of diabetes self-management education revealed that education is successful in lowering glycosylated hemoglobin levels[39].

### Diabetes self-care activities

Diabetes education is important but it must be transferred to action or self-care activities to fully benefit the patient. Self-care activities refer to behaviors such as following a diet plan, avoiding high fat foods, increased exercise, self-glucose monitoring, and foot care[40]. Decreasing the patient’s glycosylated hemoglobin level may be the ultimate goal of diabetes self-management but it cannot be the only objective in the care of a patient. Changes in self-care activities should also be evaluated for progress toward behavioral change[41].

Self-monitoring of glycemic control is a cornerstone of diabetes care that can ensure patient participation in achieving and maintaining specific glycemic targets. The most important objective of monitoring is the assessment of overall glycemic control and initiation of appropriate steps in a timely manner to achieve optimum control. Self-monitoring provides information about current glycemic status, allowing for assessment of therapy and guiding adjustments in diet, exercise and medication in order to achieve optimal glycemic control. Irrespective of weight loss, engaging in regular physical activity has been found to be associated with improved health outcomes among diabetics[42–45]. The National Institutes of Health[46] and the American College of Sports Medicine[47] recommend that all adults, including those with diabetes, should engage in regular physical activity.

### Compliance to self-care activities

Treatment adherence in diabetes is an area of interest and concern to health professionals and clinical researchers even though a great deal of prior research has been done in the area. In diabetes, patients are expected to follow a complex set of behavioral actions to care for their diabetes on a daily basis. These actions involve engaging in positive lifestyle behaviors, including following a meal plan and engaging in appropriate physical activity; taking medications (insulin or an oral hypoglycemic agent) when indicated; monitoring blood glucose levels; responding to and self-treating diabetes- related symptoms; following foot-care guidelines; and seeking individually appropriate medical care for diabetes or other health-related problems[48]. The proposed regimen is further complicated by the need to integrate and sequence all of these behavioral tasks into a patient’s daily routine.

The majority of patients with diabetes can significantly reduce the chances of developing long-term complications by improving self-care activities. Despite this fact, compliance or adherence to these activities has been found to be low, especially when looking at long-term changes. In the process of delivering adequate support healthcare providers should not blame the patients even when their compliance is poor[49]. In a study conducted among people with diabetes only 30% were compliant with drug regimens and the non-compliance was higher among the lower socioeconomic groups[50]. One of the realities about type-2 diabetes is that only being compliant to self-care activities will not lead to good metabolic control. Research work across the globe has documented that metabolic control is a combination of many variables, not just patient compliance[51, 52]. In an American trial, it was found that participants were more likely to make changes when each change was implemented individually. Success, therefore, may vary depending on how the changes are implemented, simultaneously or individually[53]. Some of the researchers have even suggested that health professionals should tailor their patient self-care support based on the degree of personal responsibility the patient is willing to assume towards their diabetes self-care management[54].

### Barriers to diabetes care

The role of healthcare providers in care of diabetic patients has been well recognized. Socio-demographic and cultural barriers such as poor access to drugs, high cost, patient satisfaction with their medical care, patient provider relationship, degree of symptoms, unequal distribution of health providers between urban and rural areas have restricted self-care activities in developing countries[39, 55–58]. In a study to identify the barriers from the provider’s perspective in regard to diabetes care factors like affordability by the patient, belief by providers that medications cannot cure patient condition, no confidence in their own ability to alter patient behavior were identified[59]. Another study stressed on both patient factors (adherence, attitude, beliefs, knowledge about diabetes, culture and language capabilities, health literacy, financial resources, co-morbidities and social support) and clinician related factors (attitude, beliefs and knowledge about diabetes, effective communication)[60].

### Recommendations for self-care activities

Because diabetes self-care activities can have a dramatic impact on lowering glycosylated hemoglobin levels, healthcare providers and educators should evaluate perceived patient barriers to self-care behaviors and make recommendations with these in mind. Unfortunately, though patients often look to healthcare providers for guidance, many healthcare providers are not discussing self-care activities with patients[61]. Health care providers should begin by taking time to evaluate their patients’ perceptions and make realistic and specific recommendations for self-care activities. Some patients may experience difficulty in understanding and following the basics of diabetes self-care activities. When adhering to self-care activities patients are sometimes expected to make what would in many cases be a medical decision and many patients are not comfortable or able to make such complex assessments. Furthermore, these requirements or modifications should be specific for each patient and should be altered depending on the patient’s response[25]. It is critical that health care providers actively involve their patients in developing self-care regimens for each individual patient. This regimen should be the best possible combination for every individual patient plus it should sound realistic to the patient so that he or she can follow it[62]. Simultaneously, health care providers should fully document the specific diabetes self-care regimen in the patients’ medical record as it will facilitate provider-patient communication and help in assessment of compliance. Also, the need of regular follow-up can never be underestimated in a chronic illness like diabetes and therefore be looked upon as an integral component of its long term management.

### Implications for practice

A clinician should be able to recognize patients who are prone for non-compliance and thus give special attention to them. On a grass-root level, countries need good diabetes self-management education programs at the primary care level with emphasis on motivating good self-care behaviors especially lifestyle modification. Furthermore, these programs should not happen just once, but periodic reinforcement is necessary to achieve change in behavior and sustain the same for long-term. While organizing these education programs adequate social support systems such as support groups, should be arranged.

### Implications for future research

As most of the reported studies are from developed countries so there is an immense need for extensive research in rural areas of developing nations. Concurrently, field research should be promoted in developing countries about perceptions of patients on the effectiveness of their self-care management so that resources for diabetes mellitus can be used efficiently.

## Conclusion

To prevent diabetes related morbidity and mortality, there is an immense need of dedicated self-care behaviors in multiple domains, including food choices, physical activity, proper medications intake and blood glucose monitoring from the patients. Though multiple demographic, socio-economic and social support factors can be considered as positive contributors in facilitating self-care activities in diabetic patients, role of clinicians in promoting self-care is vital and has to be emphasized. Realizing the multi-faceted nature of the problem, a systematic, multi-pronged and an integrated approach is required for promoting self-care practices among diabetic patients to avert any long-term complications.

## Funding

No sources of support provided.
